# Epididymal Leiomyosarcoma: One Case Report

**DOI:** 10.1155/2012/236320

**Published:** 2012-04-02

**Authors:** Mohammed Fadl Tazi, Ahmed Amine Bouchikhi, Youness Ahallal, Soufiane Mellas, Jalaleddine Elammari, Abdelhak Khallouk, Mohammed Jamal Elfassi, Moulay Hassan Farih

**Affiliations:** ^1^Department of Urology, Hassan II Teaching Hospital, Fes, Morocco; ^2^Department of Anatomy, FMPF, University of Fes, Fes, Morocco

## Abstract

Epididymal leiomyosarcoma is very uncommon. Only 16 cases have been reported in the literature. There has never been a reported case of a patient with an African origin with this tumor. We report the medical history of a 70-year-old man who presented an enormous mass located in his epididymis. A tricut biopsy was performed which allowed histological confirmation of epididymal leiomyosarcoma after which the patient underwent excision of the mass. The patient died after the first round of chemotherapy.

## 1. Introduction

Leiomyosarcoma is a tumor that can arise from any organ that contains smooth muscles. Its development in the epididymus is rare. 

Early diagnostics of this tumor have proven to be of good prognosis as many studies have showed. 

Surgical resection of the tumor associated with adjuvant chemotherapy seems efficient in its treatment. 

We report a case of a 70-year-old man who was diagnosed of leiomyosarcoma of the epididymis treated by excision of the mass followed by the 1st round of chemotherapy before his death.

## 2. Case Presentation

**Figure 1:**
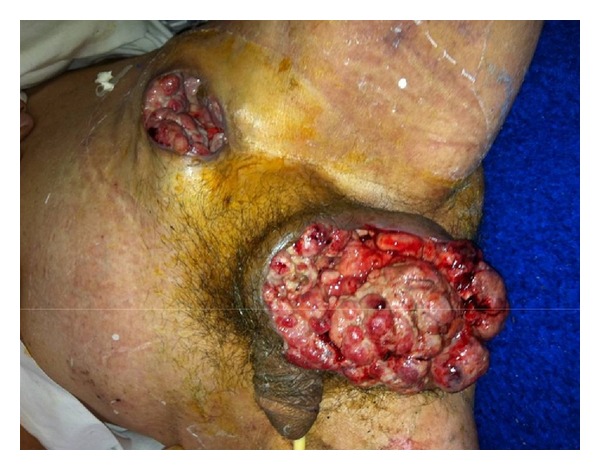
An ulcerovegetant mass with an enlarged fistulised inguinal lymph node.

A 70-year-old Moroccan male with no medical history, presented with a scrotal mass located in the left testicle and along the spermatic cord which increased in volume gradually. Medical examination showed a patient with an altered general state and an ulcerovegetant mass with its largest diameter measuring 25 cm associated with an enlarged fistulised inguinal lymph node (Figure 1). 

The dosage of tumor markers (HCG, alpha foetal protein, and PA) was negative. 

**Figure 2:**
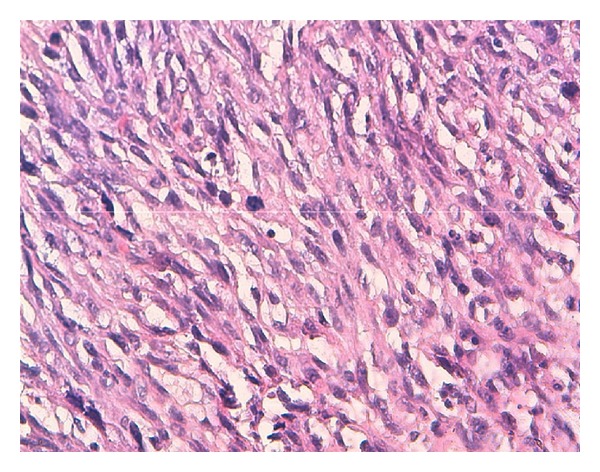
Pathology reports atypic (HES ×400).

A tricut biopsy was realized followed by histological study of the masss which confirmed an epididymal leiomyosarcoma (Figure 2). 

The patient underwent excision of the mass followed by the first round of chemotherapy, and he passed away after a month.

## 3. Discussion

Leiomyosarcoma of paratesticular tissues is very uncommon. It arises from smooth muscles which form the muscles of the scrotal sac, the seminiferous tubules, and the vascular tissues of the spermatic cord(). Its principal location is in the testicular walls and the spermatic cord [1].

Epididymal leiomyosarcoma occurs in men with ages between 50 and 80 years. It may also occur in children as there has been a reported case in a 6-year-old child [2]. 

Certain predisposing factors have been described in the literature leading to testicular leiomyosarcoma such as exposure to large doses of anabolising steroids [3], chronic inflammation [4], or past radiotherapy.

There are no reported predisposing factors leading to epididymal leiomyosarcoma with respect to its prognosis in the literature. 

It is difficult to define aggressive forms of this tumor due to its relatively rare occurrence; however, its aggressivity seems less important with respect to its location, as there has only been a single case reported of recurrence and of metastasis. The lack of a universal histological classification contributes to the difficulty in determining histological characters that influence the prognosis of this tumor. 

The only character described frequently is the mitotic index which is often elevated. Nevertheless, it seems not to help predict the agressivity of the tumor as well as its prognosis as it is not strictly related to the recurrence of the tumor nor the occurrence of metastasis [5].

Histological studies of the mass biopsied showed elevated mitotic index.

Immunohistochemical studies showed differentiated smooth muscle cells that tested positive for actin, desmin, and caldesmon. The diagnosis of leiomyosarcoma was made upon these findings.

Most authors prefer treatment by orchidectomy only [6]. An adjuvant therapy was realized in two cases: chemotherapy adjuvant in the first case (due to elevated mitotic index) [7] and radiochemotherapy in the second case [8]. 

Some authors reported that an adjuvant therapy may be performed in cases where there exist elevated mitotic index. However, it is difficult to determine the benefits of this complementary treatment as few cases have been reported in the literature [9].

## 4. Conclusion

The epididymis represents a rare location for leiomyosarcoma although its histological characteristics are the same as that found in other organs.

There is no morphological criteria that determine its prognosis (relating to its aggressivity) as well as an anatomo-pathological classification that will help to grade this tumor.

In these present times, information from the literature shows that radical orchidectomy seems to be the underlying treatment for this tumor. There is no proven efficacy for any adjuvant therapy.
